# An exercise physiologist's guide to metabolomics

**DOI:** 10.1113/EP091059

**Published:** 2024-02-15

**Authors:** Daniel J. Owens, Samuel Bennett

**Affiliations:** ^1^ Research Institute of Sport and Exercise Science (RISES) Liverpool John Moores University Liverpool UK; ^2^ Center for Biological Clocks Research, Department of Biology Texas A&M University College Station Texas USA

**Keywords:** exercise physiology, metabolism, metabolomics

## Abstract

The field of exercise physiology has undergone significant technological advancements since the pioneering works of exercise physiologists in the early to mid‐20th century. Historically, the ability to detect metabolites in biofluids from exercising participants was limited to single‐metabolite analyses. However, the rise of metabolomics, a discipline focused on the comprehensive analysis of metabolites within a biological system, has facilitated a more intricate understanding of metabolic pathways and networks in exercise. This review explores some of the pivotal technological and bioinformatic advancements that have propelled metabolomics to the forefront of exercise physiology research. Metabolomics offers a unique ‘fingerprint’ of cellular activity, offering a broader spectrum than traditional single‐metabolite assays. Techniques, including mass spectrometry and nuclear magnetic resonance spectroscopy, have significantly improved the speed and sensitivity of metabolite analysis. Nonetheless, challenges persist, including study design and data interpretation issues. This review aims to serve as a guide for exercise physiologists to facilitate better research design, data analysis and interpretation within metabolomics. The potential of metabolomics in bridging the gap between genotype and phenotype is emphasised, underscoring the critical importance of careful study design and the selection of appropriate metabolomics techniques. Furthermore, the paper highlights the need to deeply understand the broader scientific context to discern meaningful metabolic changes. The emerging field of fluxomics, which seeks to quantify metabolic reaction rates, is also introduced as a promising avenue for future research.

## Abstract (highlights)

- **What is the topic of this review?**
  The integration of metabolomics in exercise physiology.
- **What advances does it highlight?**
  Technolgical advances in the study of metabolites, with particular relevance to exercise.

## 1 INTRODUCTION

Exercise significantly challenges the body's homeostatic balance, necessitating a rapid and substantial increase in ATP resynthesis. Consequently, the rate of various bioenergetic reactions undergoes rapid alterations at the onset of exercise, and these changes in reaction rates modify the concentrations of metabolites in biofluids (including blood) and tissues like skeletal muscle. Such alterations in metabolite concentrations indicate the mobilisation, utilisation and transformation of energy substrates, notably carbohydrates and lipids, to fulfil the increased ATP requirements of working muscle. The array of metabolites produced by bioenergetic pathways also serves as signals transduced to molecular events (Febbraio et al., 2000), leading to the transactivation of genes and the activation/suppression of both protein synthesis and degradation. Examples of some metabolites that have been established in response to muscle contractile activity and act as crucial chemical signals that stimulate adaptation are AMP, the ADP:ATP ratio, Ca^2+^, creatine phosphate, succinate, lactate, glycogen, fatty acids, NAD and NADH, and amino acids (Baker & Rutter, 2023; Hawley et al., 2006; Reddy et al., 2020; Sancak et al., 2008).

On a background of exercise, nutritional status adds further complexity to metabolic regulation. Exercise performed in the fasted state increases lipolysis and the utilisation of lipids as an energy source, but after feeding carbohydrates, fatty acid entry to the mitochondria is compromised, and carbohydrate oxidation predominates (Febbraio et al., 2000; Horowitz et al., 1997). This is then reflected in the metabolites produced as a consequence of fatty acid or carbohydrate metabolism. Taken together, exercise metabolism represents one of the most powerful examples of the dynamic nature of the metabolome in response to a stimulus.

Since the seminal works of pioneering exercise physiologists August Krogh, Archibald V. Hill and Per‐Olof Åstrand, technology has revolutionised how data are collected, analysed and interpreted. In early exercise physiology research, biochemical analyses were predominantly targeted analyses of biofluids, such as blood, urine and saliva, collected from exercising participants, typically limited to a very small number of metabolites per assay. Indeed, August Krogh had an exceptional ability to design and build equipment to assess biochemical variables with great precision, albeit limited to a small number of variables at a time (Larsen et al., 2021). Since exercise induces organism‐level stress, capturing the complexity and interconnectedness of metabolic pathways and networks is essential, providing a systems‐level understanding of biological processes. The ability to detect metabolites in biofluids and tissues has benefitted significantly from the advancements, accessibility and usability of technology.

Metabolomics, a field of scientific study focusing on the comprehensive analysis of metabolites within a biological system, is a prime example of an area that has benefitted from technological and bioinformatic advancements. Metabolomics is an increasingly common analytical approach in many areas of biological research and is gaining popularity in the exercise physiology domain. Metabolomic studies involve identifying, quantifying and characterising metabolites in biological samples, including blood, urine, tissues and cells. As metabolites are both intermediates and end products of cellular processes, they provide a biochemical ‘fingerprint’ of cellular activity, revealing the metabolic pathways and processes occurring within an organism and broadening the analytical lens beyond single‐metabolite assays. The development of high‐throughput techniques such as mass spectrometry (MS) and nuclear magnetic resonance (NMR) spectroscopy has dramatically enhanced the speed and sensitivity of metabolite analysis. These techniques allow for the simultaneous detection and quantification of numerous metabolites in a single biofluid or tissue extract. With increased accessibility to these technologies and bioinformatics tools and expertise, the field of exercise physiology has seen a sharp increase in research papers utilising omics approaches (Schranner et al., 2020). However, in many cases, poor study design, lack of data quality control, erroneous statistical approaches and incorrect data interpretation pose significant challenges to advancing the field.

This review was written as a guide for exercise physiologists to assess whether a metabolomics approach is appropriate for their research design and to facilitate better study design, data analysis and interpretation, manuscript preparation and peer review. As such, the authors intend to provide a practical guide to readers, whilst comprehensive reviews and meta‐analyses on metabolomics can be found elsewhere (Alseekh et al., 2021; Dunn et al., 2011; Sakaguchi et al., 2019; Wishart et al., 2022).

## 2 SHOULD YOU USE METABOLOMICS IN YOUR RESEARCH DESIGN?

**FIGURE 1:**
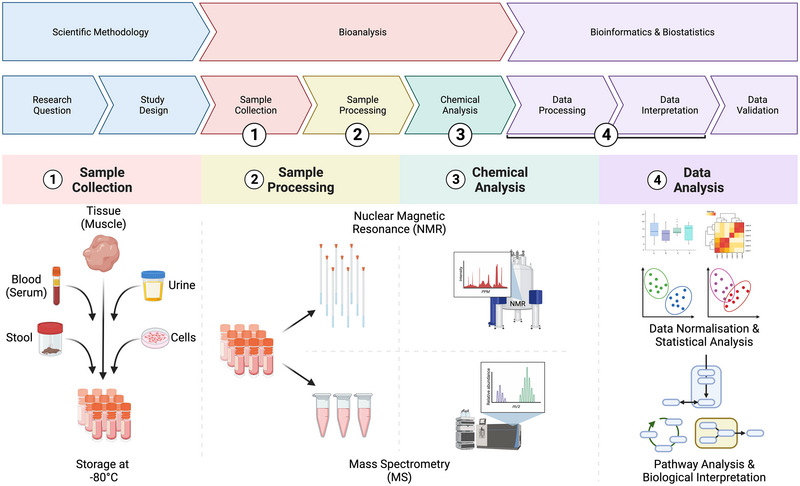
Overview of a typical metabolomics study. Following the generation of a hypothesis and subsequent research design, samples require collection (step 1) before undergoing specific sample preparation, including metabolite extraction, depending on the analytical platform of choice and the metabolites that the researcher intends to characterise (step 2). A specialist facility or technician usually performs chemical analysis by ^1^H‐NMR or mass spectrometry (step 3). Once complete, spectral processing, data normalisation and statistical analysis are conducted ideally with the support of a bioinformatician, including pathway analysis to assist with biological interpretation (step 4).

Whether or not metabolomics is adopted as an experimental approach depends on the research question and an understanding of what metabolomics can offer the researcher when performed properly. In later sections of this review, we present examples of insights gleaned from metabolomics in exercise physiology research. What these examples demonstrate is that better insights into the connectedness and interplay between metabolic pathways can be captured by metabolomics than by sampling individual metabolites. When designing investigations in exercise physiology and nutrition, metabolomics can be particularly potent, delivering a snapshot of metabolic pathway alterations that result from these conditions. However, the metabolome is highly sensitive and dynamic, so researchers must be aware that external factors such as age, circadian rhythm and gut microbiota composition can significantly influence metabolomic profiles (Holmes et al., 2008). Proper experimental design, including careful selection of controls and consideration of confounding variables, is paramount. In essence, while metabolomics can be a robust tool for dissecting functional implications in a biological system, its utility hinges on the appropriateness of its application to the research question at hand. Figure 1 outlines a macroscopic overview of a typical metabolomics workflow. Key aspects of this workflow will be discussed in detail in subsequent sections of this review.

## 3 SCIENTIFIC METHODOLOGY

### 3.1 The research question and the importance of careful study design

The use of metabolomics should be carefully considered depending on the research question. In general, omics approaches are excellent for broad, exploratory research where systems‐based insights are required. However, if the research question is very specific, targeting a particular metabolite or pathway, then a targeted approach may be more efficient and cost‐effective. Omics studies generate vast amounts of data, requiring complex tools for analysis and interpretation, as discussed later in this guide. This complexity can be a barrier if there is not sufficient expertise or computational resources available. Additionally, the sheer volume of data can sometimes obscure rather than clarify the understanding of physiological processes. It is also important to consider that whilst metabolomics approaches are useful for understanding the breadth of molecular changes, they often don't provide direct insight into the functional significance or mechanistic pathways. Therefore, researchers should be aware that complementary experimental approaches are usually needed to understand how these changes impact physiological functions. Later in this guide, we explore some published evidence where metabolomics has been utilised and has provided useful insights in different contexts of exercise physiology research.

**FIGURE 2:**
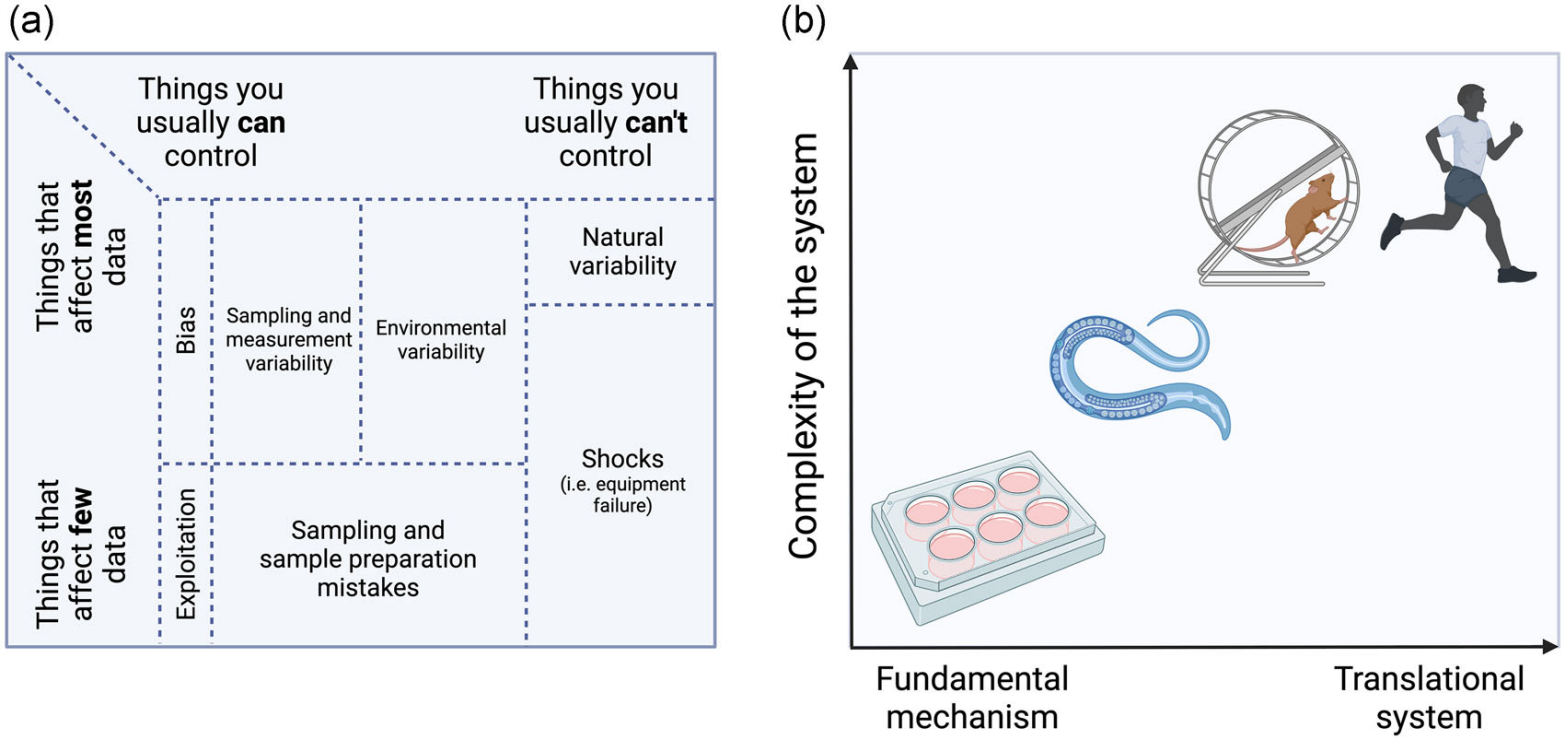
Considerations for designing metabolomics experiments. (a) Common sources of variation in metabolomics data that can and cannot be controlled by the researcher. (b) The trade‐off between the complexity of the system and the translational potential of that system.

If metabolomics is to be utilised, the study design is a critical consideration when metabolomics is identified as a key outcome measure at this stage, rather than ex post facto inclusion with surplus sample. In this way, critical experimental variables that affect sample quality and experimental bias can be factored into the study design. In Figure 2a, sources of variation in metabolomics experiments are outlined. Consequently, the more control over the controllable sources of variation, the lower the sample size required for the study. As depicted in Figure 2b, in vitro models offer the greatest degree of control, and therefore only relatively small sample sizes are typically required. Whilst exercise physiologists may opt for in vitro studies to elucidate fundamental mechanisms and, in some cases, rely on animal models to perform genetic manipulations or unfeasible experimental methods for human research, ecologically valid human exercise studies are the gold standard for translational potential (i.e., the capacity to inform practice). As such, larger sample sizes are required for human trials compared to simple model organisms and in vitro systems to account for inherent individual variability.

Considering the sources of variability described in Figure 2, we provide a checklist (Table 1) that could be implemented in the design of exercise physiology experiments when a metabolomics approach is used. Consistency is critical in metabolomics research. Every stage of the experimental workflow, from sample collection to data analysis, must be well planned and executed to minimise variation and maximise the reliability of findings. This checklist may help researchers limit unwanted variation sources and maximise the experimental condition's metabolic phenotype. Of particular importance to exercise physiologists is the issue of sample timing. In a systematic review of exercise metabolomics studies, Schranner et al. (2020) demonstrates the number of metabolites that are changed with the time point of sampling. The authors categorised time points as early changes, within 0.5 h after exercise; intermediate changes, between >0.5 and 3 h after exercise; and late changes, between >3 and 24 h after exercise. In early and intermediate sampling instances, 38 metabolites, predominantly amino and fatty acids, showed changes not observed in late sampling experiments. The changes exclusive to early sampling were primarily characterised by 19 amino acids, followed by 11 short‐ and medium‐chain acylcarnitines, six carbohydrates, and intermediates of the TCA cycle. Conversely, the intermediate changes comprised diverse metabolite groups, including amino acids, nucleotides, vitamins/cofactors and xenobiotics. As such, if performing untargeted metabolomics then researchers should be aware of the limitations of collecting a single time point following an intervention. Conversely, time points should be chosen carefully if a targeted panel of metabolites is to be profiled.

**TABLE 1:** Exercise metabolomics study design checklist to help minimise sources of unwanted variation and maximise the signal: use this checklist to ensure that samples arrive at your chosen metabolomics facility in an optimal condition to maximise metabolite detection.

Source of bias or variation	Solutions
Experimental planning phase	
Sample heterogeneity	○ Account for potential differences due to age, gender or developmental stage ○ Consider circadian rhythms – aim to collect samples at a consistent time of day ○ Collect the correct type of sample based on the metabolites you wish to detect
Experimental bias	○ Plan for blinding during sample collection, analysis and data interpretation to reduce bias. Where possible, use a placebo‐controlled, randomised controlled trial
Variability in lifestyle and dietary factors	○ Record or control dietary intake prior to sample collection ○ Control or account for factors such as exercise (i.e., within 24–48 h prior to participation in the trial), sleep and stress levels ○ Ensure exclusion criteria are specific enough to exclude those taking medications and supplements that would interfere with the metabolome or other study outcomes
Underpowered sample	○ Ensure that each experimental group includes multiple biological replicates ○ Understand the inherent biological variability to determine appropriate sample size

Sample collection phase	
Sample collection errors	○ Use standardised methods for sample collection across all participants, regardless of their group assignment. ○ Minimise the time between sample collection and freezing to minimise changes in metabolites that have occurred during exercise. If handling multiple types of samples and collecting other data, is there another team member that could specifically process samples to reduce time between collection and freezing? ○ Ensure consistency between participants in sample collection time relative to the exercise stimulus. ○ Collect duplicate samples or split samples in case of errors or shocks to a sample. ○ Choose correct collection vessel for samples. For example, if collecting serum or plasma, blood collection tubes without additives like EDTA should be used.
Sampling bias	○ Ensure blinding during sample collection, analysis, and data interpretation to reduce bias. Practically, this can be achieved by having a separate team handle the allocation and labelling of samples, using codes that are not decipherable by the collection team
Environmental variability	○ Control laboratory conditions during experiments to minimise environmental impact. This may not be feasible for field‐based sample collection. In which case see next bullet ○ Note any environmental changes (e.g., temperature, humidity) that might impact samples

Sample processing	
Variability in sample matrix	○ If collecting different biofluids, confirm that all samples are of a similar biological matrix (e.g., plasma, serum, urine) before proceeding to extraction
Pipetting errors	○ If considered during data collection, duplicate samples or split samples should be available
Incorrect extraction method for metabolites of interest	○ Confirm the extraction method is correct. Work with ^1^H‐NMR or MS technician to determine the best approach for your workflow
Errors during extraction	○ Ensure high‐quality, clean solvents and laboratory materials are used ○ Ensure solvents are the correct temperature when added to the samples ○ Keep the extraction conditions consistent across all samples within a study ○ Conduct the extraction process as rapidly as possible to minimise degradation ○ Store samples properly before and after extraction to prevent degradation or changes in metabolite composition

In all circumstances, the time taken between the collection of the sample and processing and freezing the sample should be kept consistent and to minimum. Others have reported on the response of the human plasma metabolome to common preanalytical variations including prolonged processing times at different temperatures (Kamlage et al., 2014). Samples kept at room temperature for 1 h resulted in a 22% change the metabolome, whilst storage on wet ice for 2 h and 6 h resulted in a 16% and 17% change, respectively. From the authors’ experience, sample collection and processing immediately following the exercise stimulus can be challenging, so optimising the laboratory set‐up is critical to successful sample collection.

## 4 BIOANALYSIS

### 4.1 Preparing samples for analysis

Depending on the research question and the metabolites the exercise physiology researcher is interested in detecting, sample processing differences must be considered. Unlike the proteome or transcriptome, the metabolome is highly chemically diverse, and as such, no single method can capture all metabolites. In some cases, metabolite extraction may need to be performed before getting the samples to the analytical facility, and as such, we detail some key points here. In other cases, the facility may perform metabolite extraction for the researcher.

The selection of an extraction method in metabolomics is largely determined by the sample type, such as biological fluids, tissues, or cells, and the metabolites of interest. In NMR metabolomics, various standard methods are employed. These include solvent extraction, which is suitable for polar metabolites like amino acids, sugars and organic acids. For a broad spectrum of lipids such as fatty acids, sterols and glycerolipids, methods like Folch or Bligh and Dyer extraction are used. Solid‐phase extraction is utilised for isolating specific compounds like phenolic compounds and alkaloids, or lipid types. Ultrafiltration allows for the separation of small molecules from larger ones. Additionally, cold extraction techniques are essential for heat‐sensitive metabolites.

From the authors’ experience, using acetonitrile and doubly distilled water for extracting metabolites from biofluids yields good coverage of various metabolites. In contrast, MS metabolomics demands more varied approaches due to its sensitivity and specificity. Techniques such as liquid–liquid extraction are applied to separate mixtures into polar and non‐polar fractions, which is crucial for analysing targeted lipids and water‐soluble metabolites. Protein precipitation is particularly effective for biofluids like plasma or serum, as it helps remove proteins and isolates target metabolites like small peptides, amino acids and organic acids. Methanol–water extraction is versatile, extracting a wide range of metabolites from polar compounds to moderately polar lipids. Acetonitrile–water extraction is another method used for biofluids, recovering a broad spectrum of metabolites including small organic acids and lipids.

For further detailed information on metabolite extraction methods, readers are referred to the comprehensive paper by Barnes et al. (2016). Additionally, consulting with a technical specialist at an analytical facility is recommended to determine the most appropriate extraction procedure for specific research needs.

### 4.2 What type of measurement tool is right for your metabolomics research?

The tools to perform metabolomics research have been available since the 1950s (Williams, 1951) and publications reporting the simultaneous quantification of urinary metabolites appeared in the 1970s (Horning & Horning, 1971; Pauling et al., 1971). The two metabolite profiling methods (see Figure 1, Step 3) established during this era were MS coupled with chromatography methods and proton (^1^H)‐NMR spectroscopy. These approaches form the basis of the two main metabolomics techniques used today, albeit having benefitted from considerable technological advances.

Both MS and NMR applications can perform targeted and untargeted metabolomics (see Table 2). Targeted metabolomics involves the selective analysis of a predefined set of known metabolites. In this approach, researchers specifically target and measure a set of metabolites of interest based on prior knowledge or specific hypotheses. The analytical methods used in targeted metabolomics are designed to detect and quantify these predefined metabolites accurately. Targeted metabolomics is commonly used in hypothesis‐driven research, where researchers have a specific question or metabolic pathway of interest. It allows for precise quantification of known metabolites and can provide insights into targeted metabolic processes.

**TABLE 2:** Critical features of targeted and untargeted metabolomics approaches.

Targeted	Untargeted
Specificity: targeted approaches focus on a limited number of metabolites or a specific metabolic pathway or class of metabolites	Broad coverage: untargeted approaches aim to capture a wide range of metabolites, including known and unknown compounds
Quantitative analysis: targeted metabolomics aims to measure metabolite concentrations accurately	Semi‐quantitative analysis: while quantification is possible in untargeted metabolomics, the primary focus is on relative abundance comparisons rather than absolute quantification
Known metabolites: the selection of metabolites for analysis is based on prior knowledge or specific research objectives	Discovery‐oriented: untargeted approaches allow for the identification of novel metabolites and unexpected metabolic pathways
Lower coverage: targeted approaches provide detailed information about specific metabolites but may not capture the full breadth of the metabolome	Hypothesis‐generating: untargeted approaches generate large datasets, and the identification and interpretation of metabolites require extensive data analysis and bioinformatics

Untargeted metabolomics involves a comprehensive and unbiased analysis of all detectable metabolites within a biological sample without prior knowledge of the specific metabolites present. It aims to capture a global view of the metabolome and identify as many metabolites as possible without bias. Untargeted metabolomics is often used in exploratory research or when the metabolic landscape of a sample is not well‐known. It can reveal metabolic alterations, identify potential biomarkers, and provide a more comprehensive understanding of metabolic pathways and networks.

From a practical exercise physiology perspective, targeted metabolomics may be more appropriate when researchers wish to study a specific metabolic pathway of interest, validating underlying mechanisms by which a specific metabolite is changed during hypothesis‐generating experiments. For example, metabolomics approaches could be used to interrogate the time‐dependent impact of exercise on metabolism (Bennett & Sato, 2023). Untargeted multi‐tissue metabolomics of mouse tissue harvested at 4‐h intervals over 24 h identified 2‐hydroxybutyrate (2‐HB), a ketone body, as a circadian oscillating metabolite (Dyar et al., 2018). Immediately following 1 h of treadmill exercise, 2‐HB was substantially increased in mouse skeletal muscle during the early active phase but not following exercise in the early rest phase (Sato et al., 2022). To validate the role of 2‐HB as an exerkine (exercise inducible compound with signalling roles within and between tissues), a targeted metabolomics approach was implemented using gas chromatography–mass spectrometry (GC‐MS) to quantify the impact of exogenous 2‐HB (via injection) on whole body metabolism and associated metabolites in mice (Sato et al., 2022). Unlike the previous untargeted approach, biological samples were analysed with known standards to improve the quantification accuracy for several metabolites of interest. The authors reported that circulating 2‐HB transiently reduced energy expenditure and altered substrate utilisation time‐dependently, increasing glycaemia and altering liver and muscle amino acid metabolism. Considered an early biomarker of type‐2 diabetes, 2‐HB may be a crucial target for treating metabolic disease.

Conversely, Pugh et al. (2021) employed untargeted ^1^H‐NMR metabolomics to explore the effects of probiotic supplementation on the human serum and skeletal muscle metabolome following a competitive marathon. This study identified a potential protective effect of the major metabolic perturbations induced by marathon running by consuming a multi‐strain probiotic 4 weeks before the marathon. As an example of good practice in exercise metabolomics research design, we did the following to minimise unwanted bias: randomly allocated participants to placebo or probiotics in a blinded fashion. Following the supplementation protocol, we prescribed a 24‐h pre‐race diet and remote food photography to verify participant adherence, provided a pre‐race meal in line with best practice fuelling guidance, supplied and monitored carbohydrate intake during the marathon, and collected samples before breakfast and immediately after the marathon by situating blood collection and biopsy facilities directly adjacent to the running track. Centrifuges and freezers were also nearby to facilitate rapid sample processing and storage.

Since both NMR and MS can be used to perform targeted and untargeted metabolomics, a common question the authors are asked is, which of these techniques should be used? Both techniques have merits and weaknesses; neither approach can obtain complete metabolome coverage. It is noteworthy, however, that both ^1^H‐NMR and MS can be used simultaneously to overcome some of the limitations of each method when used in isolation. In keeping with the practical nature of this review, in Table 3 we provide a simple comparative breakdown of ^1^H‐NMR and MS metabolomics applications. For an extensive overview of these two analytical approaches, readers are referred elsewhere (Wishart et al., 2022).

**TABLE 3:** Comparative breakdown of ^1^H‐NMR and MS metabolomics applications.

Criteria	Mass spectrometry	^1^H‐NMR
Sample preparation	Requires extensive preparation, which can be time‐consuming and may introduce bias	Minimal preparation needed since NMR provides an overall profile of all proton‐containing metabolites, including lipids, in a sample without the need for prior separation or specific ionisation techniques
Analysis destructiveness	Destructive, samples cannot be reused	Non‐destructive, allowing for further analysis of the same sample
Sensitivity and specificity	High sensitivity and specificity, able to detect low abundance metabolites	Less sensitive compared to MS, may not detect very low abundance metabolites
Structural elucidation	Very effective for determining molecular weight and formula	Provides detailed structural information, including stereochemistry
Quantitative analysis	Absolute quantification usually requires extensive calibration with standards	Easier to perform absolute quantification without the need for multiple standards
Throughput for comparative studies	High‐throughput capability, suitable for large‐scale studies	High‐throughput capability and superior reproducibility, suitable for large‐scale studies
Reproducibility	Can be highly reproducible but requires careful method standardisation	Highly reproducible, given that the sample handling and preparation are less complex
Coverage	Can cover a broad range of metabolites with good sensitivity, including those that are semi‐polar and non‐polar	More uniform coverage for metabolites that are present in concentrations above its detection limit but may miss low abundance metabolites that MS can detect
Data complexity	Generates complex data that require extensive processing and analysis	Generates relatively simpler spectra, which are easier to interpret but spectral processing can be challenging when metabolites overlap
Sample volume	Often requires small volumes, which can be advantageous when sample availability is limited	Requires more substantial sample volumes, which might be a limitation with scarce samples
Cost	Can be costly due to consumables, maintenance and operation requirements	Generally lower operational costs and less maintenance intensive

## 5 DATA ANALYSIS

### 5.1 Data pre‐processing, quality control and quality assurance

There are differences in data pre‐processing dependent on whether targeted or untargeted metabolomics is performed. In targeted metabolomics, during the assay development, lower and upper limits of quantification are determined for each metabolite (LLOQ and ULOQ, respectively) using reference samples such as the National Institute of Standards and Technology (NIST) standards. These quality control (QC) samples determine the coefficient of variation of measured samples from the researcher's experiment. Since batch effects are common in metabolomics raw data sets, reference samples can also be used to normalise batch effects. For more details on the targeted metabolomics QC process, readers are referred to Dyar et al. (2019).

**FIGURE 3:**
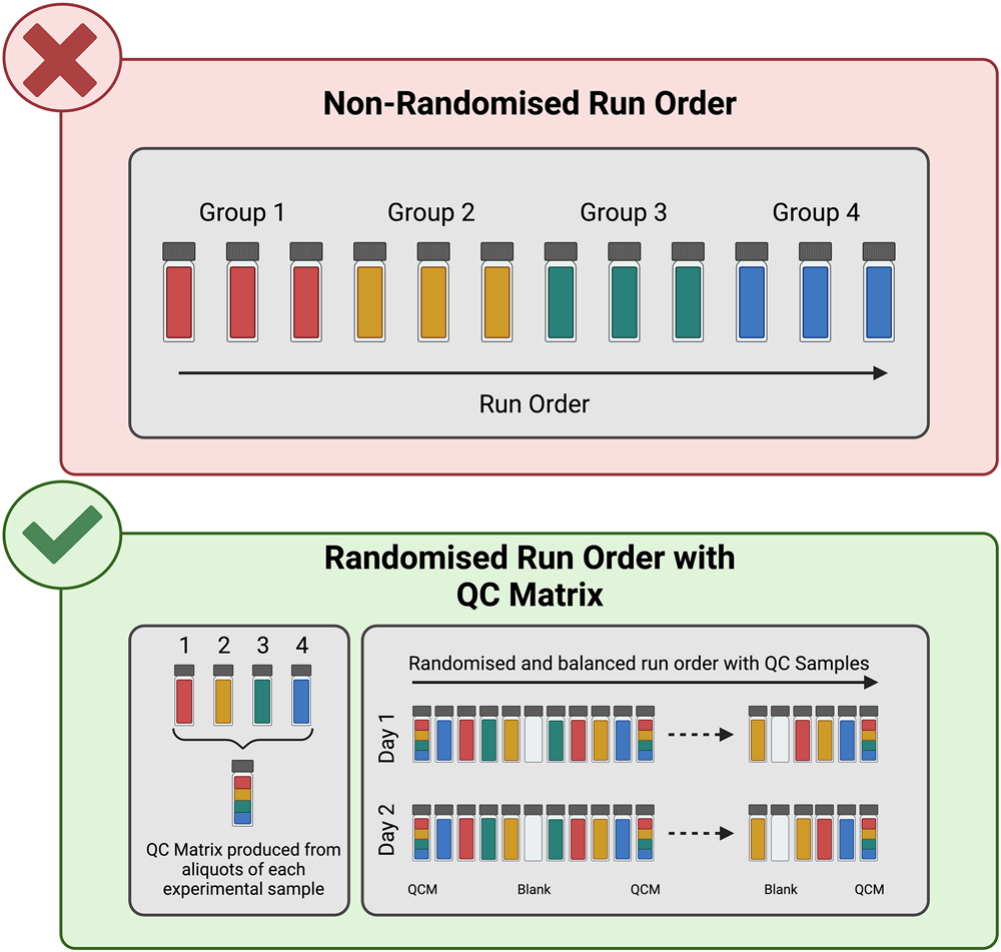
Sub‐optimal, grouped sample run order (top) and optimal sample run order within randomised sample order across each run and balanced between days, including quality control matrix (QCM). Comprising a small aliquot of each sample, the QCM is periodically sampled throughout data acquisition and allows the calculation of within and between runs variability based on consistently measured biochemicals across each run. Blank samples could contain either ultra‐pure water or solvents used during metabolite extraction, allowing the quantification of baseline signals and identifying potential sources of contamination, respectively.

In the case of untargeted metabolomics, the QC process is different since it is not feasible to have reference samples for all the metabolites that might be detected in an experimental sample. In this case, QC samples derived from pooled aliquots of all biological samples in a study serve as a consistent analytical reference when processed alongside actual samples (Figure 3). Their primary role is to gauge the platform's analytical stability and variability over time, with consistent metabolite profiles in these QC samples underpinning the dependability of the entire analysis. Additional quality control practices are employed to ensure data accuracy and reliability in targeted and untargeted metabolomics. Blank samples, consisting of all components except the actual biological sample, such as solvents or extraction buffers, detect potential contaminants or artefacts in the analytical procedure. To sidestep issues related to time or batch effects during analysis, especially in extended runs, samples are not processed in their collection sequence; instead, a randomisation approach is adopted. Additionally, analysing the same sample multiple times (i.e., technical replicates) is crucial for determining data reproducibility and identifying any inconsistencies in the data.

After data acquisition, quality assurance (QA) comes from metrics like retention time shifts, peak width and signal‐to‐noise ratios. These metrics ensure that the acquired data meets the desired quality standards and track any potential drifts or anomalies in the dataset before progressing to data analysis. Data QC and QA are critical examples of where a trained collaborator is highly recommended to help exercise physiologists ensure their data pass QA checks.

### 5.2 Metabolite annotation

Those new to metabolomics may wonder how to get from an analytical platform to data that can be used for statistical analysis and biological interpretation. The authors preface the following advice with a strong recommendation to having a team member or collaborator assist with data analysis. Undoubtedly, one of the most challenging aspects of performing a successful metabolomics experiment is data processing. Although easy‐to‐use analytical packages are available, it is crucial to understand what those packages are doing and verify all steps have been performed correctly.

A significant challenge for both ^1^H‐NMR and MS metabolomics is metabolite annotation. As described previously in extensive detail (Viant et al., 2017), interpreting metabolomics data with confidence and making substantive biological conclusions is contingent upon the ability to assign specific structures to detected peaks (whether they be ^1^H‐NMR or MS peaks), effectively labelling them as known metabolites. Discussing exercise metabolomes depends on the metabolites determined in study samples. In many cases, metabolomics analyses will report numerous ‘unknown’ peaks, which may reflect the database used to annotate the spectra or that the peak has not yet been characterised as a metabolite. Exercise physiology researchers must be aware of the current limitations in metabolite annotation and scrutinise the approach they are taking in their research or when reviewing manuscripts from other researchers.

First, it is essential to understand that whether the researcher employs NMR or MS‐based metabolomics, a successful experiment will produce raw data as spectral peaks. While MS and NMR spectroscopy produce spectral peaks, these peaks are fundamentally different. MS characterises metabolites based on their mass‐to‐charge ratio (*m*/*z*), yielding spectra that reflect molecular weight and, when using tandem MS, infer structural elements from fragmentation patterns. On the other hand, NMR offers insights based on resonance frequencies of atomic nuclei within a magnetic field, revealing detailed structural information about the molecule. In each case, metabolite identities must be assigned to these peaks. Whilst NMR or MS may be powerful enough to detect many ‘features’ in the researchers’ samples, assigning metabolites depends on a good reference database to which those features can be given an identity. Several efforts are being made to improve metabolite annotation; for example, in NMR metabolomics, the Collaborative Computational Project for NMR (CCPN; https://ccpn.ac.uk) aims to improve and integrate software tools for scientists involved in NMR spectroscopy of biological molecules. It is beyond the scope of this review to discuss the different methods for assigning metabolite identities to spectral peaks, and readers are directed elsewhere to learn more (Rosato et al., 2018). Additionally, standardised quality control procedures and reporting standards have been recommended by the Metabolomics Standards Initiative (MSI) (Salek et al., 2013; Sumner et al., 2007) to help researchers ensure consistent and accurate reporting of all metabolomics data to facilitate comparisons between studies.

### 5.3 Statistical analyses

While a specialist team member or collaborator may perform sample analysis, data pre‐processing and QC, with metabolites identified, the exercise physiologist will often take the reins when it comes to data analysis and interpretation. Below, we highlight some basics of data analysis and critical considerations.

**FIGURE 4:**
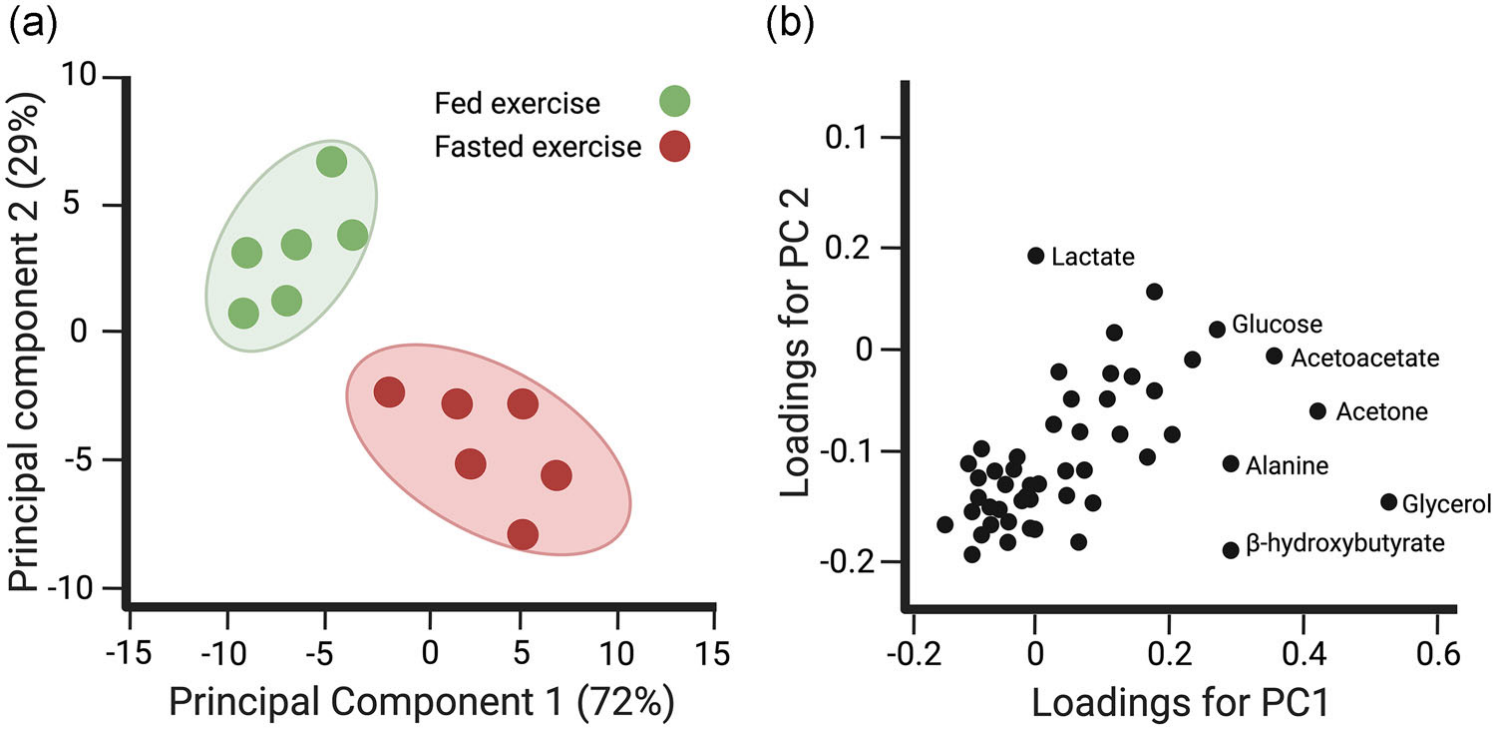
Interpreting a PCA plot and loadings plot. In the PCA scores plot (a), look for patterns, clusters and outliers. In this example of fasted vs fed exercise, PC1 explains 72% of the variance, PC2 explains 29%, and fasted and fed exercise samples cluster separately into two groups. This means PC1 might be capturing changes associated with fasted exercise. In the PCA loadings plot (b), look for variables far from the origin and those that group together. In the loadings plot example, if specific metabolites are far along PC1, those are likely the metabolites whose levels explain variance associated with fasted exercise.

Given the high dimensionality and complexity of metabolomics data, multivariate statistical approaches like principal component analysis (PCA) and partial least squares discriminant analysis (PLS‐DA) are frequently used for data analysis. PCA and PLS‐DA are typically performed before univariate statistical tests for several reasons. Interpreting a PCA plot involves understanding what the PCA plot is showing and then relating this to the context of the data. PCA plots can come in two main types, score plots and loadings plots, and both are used together to interpret the PCA results comprehensively (see Figure 4). Each axis represents a principal component, a combination of features that captures the maximum variance. The first principal component (PC1) captures the most variance, and each subsequent principal component (like PC2) captures less. The spread of the samples along the PC axes shows how they differ from one another. If samples are spread out widely along the first principal component, PC1 explains significant diversity within the data. If there are clusters of samples, it suggests that they are similar to each other in their data profile, that is, there are metabolic similarities within that group. Samples that are far away from all others may be outliers. This could suggest exceptional cases, experimental errors, or novel features warranting further investigation. PCA can also observe major sources of variation in the data, which helps identify outliers or samples that do not behave as expected or in line with other samples within the dataset. These outliers might be due to technical errors, contamination or interesting biological variations. If the samples are from different experimental groups (like fasted‐ vs fed‐exercise, for example), seeing the groups separated along a principal component can suggest that the intervention affects the variables that heavily load on that component. In other words, it suggests the samples from each group are metabolically distinct. A PCA loadings plot, on the other hand, displays how much each variable (metabolite) contributes to each principal component. Variables further from the origin contribute more to the variance captured by the principal components. This can indicate which metabolites are most important in differentiating the samples. Variables close to each other may be positively correlated, while those on opposite sides of the origin are negatively correlated. By comparing the loadings plot with the score plot, you can infer which variables are important for the separation observed in the score plot. For example, if a group of samples is separated along PC1 in the score plot, the variables farthest along PC1 in the loadings plot are those most relevant to that separation. It is important to remember that while PCA is a good tool for exploratory data analysis, the results are descriptive and not inferential, meaning that while PCA can show you patterns, it cannot prove that those patterns are statistically significant or causally relevant without further analysis.

Performing a PLS‐DA is an additional multivariate approach for understanding the underlying structure of the dataset. Unlike PCA, however, PLS‐DA is a *supervised* method and uses class‐label information to identify specific metabolites that contribute to maximising the separation between predefined classes. These classes could be treatment groups or any other categorical variable in metabolomics. The PLS‐DA can help identify metabolites that are most important for discriminating between classes. The variable importance in the projection (VIP) score is commonly used in PLS‐DA to rank metabolites based on their contribution to the model. For example, when comparing pre‐ to post‐exercise serum samples, lactate is likely to have a far greater abundance post‐exercise than pre. It would be an essential metabolite for differentiating between time points and having a high‐ranking VIP score in the PLS‐DA model. This type of analysis has limitations, and overfitting is often a concern with PLS‐DA, significantly when the number of metabolites far exceeds the number of samples. It is crucial to validate the model using cross‐validation and permutation testing to ensure its robustness and reliability. In simple terms, cross‐validation involves systematically splitting the data into training and testing sets instead of using the entire dataset to train a model and then testing on those same data (which can lead to overfitting). The model's performance is then averaged over these different splits to estimate its accuracy better. Permutation testing involves randomly shuffling the class labels (categories or groups to which data points belong) of the samples and then running the PLS‐DA model on this permuted data. This process is repeated many times. If the model gives good results on the actual data but poor results on the permuted data, it suggests that the model is genuinely capturing some structure in the data and not just fitting to random noise.

## 6 INTERPRETING YOUR METABOLOMICS DATA IN AN EXERCISE METABOLISM CONTEXT

Our current understanding of exercise metabolism is primarily based on data collected by whole‐body indirect calorimetry and targeted metabolite analyses, focusing on key substrates such as glucose, lactate, glycogen and non‐esterified fatty acids, for example. While this approach allows us to characterise the overall metabolic demands of exercise, insight into the contribution of specific energy systems and mitochondrial function is impossible (San‐Millan & Brooks, 2018). Metabolomics represents a powerful approach to comprehensively study metabolites in an exercise context, including amino acids, lipids, sugars, organic acids and other small compounds that serve as substrates, intermediates or end products of cellular metabolism (Dunn et al., 2011). Biological interpretation of metabolomics data relies on two basic steps, metabolite identification and functional analysis, which bridges the gap between raw data and meaningful biological knowledge, enabling researchers to understand cellular metabolism and its implications for exercise, nutrition and disease.

Functional analysis of a biological system requires a knowledge/database defining functionally related molecules and a statistical algorithm to perform enrichment testing (Xia, 2017). Over‐representation analysis (ORA) is one approach to developing a functional understanding of the metabolome; this approach tests whether metabolite groups appear more often than expected at random. ORA requires prior statistical comparison (such as Student's *t*‐test or ANOVA), with metabolite significance used as a criterion for inclusion in ORA. A relatively simple approach, it is criticised for its arbitrary selection of a threshold for metabolite inclusion within the analysis, as adjusting any threshold could lead to different interpretations of a dataset. Furthermore, a significance threshold requires significantly different metabolites between groups or conditions, and without this, ORA cannot be implemented.

To address the shortcomings of ORA, metabolite set enrichment analysis (MSEA) (Xia & Wishart, 2010) directly tests the enrichment of functional metabolite groups using the complete concentration data without preselection of metabolites. This allows MSEA to conduct quantitative enrichment analysis based on individual metabolite concentrations. MSEA groups molecules labelled with biologically meaningful names, making it a popular approach for omics data interpretation. However, simply grouping metabolites followed by enrichment tests ignores the interconnected and interdependent nature and the inherent overlaps/hierarchies among different groups of metabolites. For instance, changes in a primary metabolite within a pathway tend to have a larger impact on its overall functions than those downstream. Integrating functional analysis with pathway/network topology analysis will improve the accuracy of ranking the resulting biological process list.

In a notable study, San‐Millan et al. (2020) utilised MS‐based metabolomics to explore the exercise metabolome of world tour professional cyclists following an exhaustive exercise bout. They identified 355 metabolites and analysed the data using PCA and hierarchical clustering. The results highlighted metabolites central to energy metabolism, such as glycolysis and the TCA cycle, positively correlated with exercise capacity. Furthermore, the authors describe several amino acids that, when elevated before exercise, were associated with improved performance. Additionally, isoleucine, leucine and asparagine were decreased post‐exercise, suggesting preferential catabolism for ATP production, supported by increased acyl‐carnitines in the same subjects. Whilst several metabolites were identified as necessary for distinguishing performance levels in these cyclists, several further steps are required before they can be considered accurate ‘biomarkers’. Validation studies with larger cohorts across a diverse range of populations are needed to confirm the reliability and accuracy of any identified metabolites as biomarkers; given the homogeneous, highly trained nature of the study participants, the metabolomic data may not generalise to lesser‐trained individuals. Furthermore, longitudinal studies would allow researchers to monitor changes in specific metabolite level differences over time and whether their changes reflect differences in performance.

The impact of commencing exercise with low muscle glycogen availability is relatively well understood, with decreased carbohydrate utilisation with a concomitant increase in lipid oxidation relative to exercise with moderate or high glycogen availability (Hearris et al., 2019). Metabolomic analysis of serum collected before and immediately after exercise with high or low muscle glycogen availability revealed increased branch chain amino acid (BCAA), acyl‐carnitine, urea and 3‐methylhistidine, indicative of increased protein catabolism during exercise (Margolis et al., 2021). Following exercise with low muscle glycogen availability, BCAA metabolites such as 3‐methyl‐2‐oxovalerate, 4‐methyl‐2‐oxopentanoate and 3‐hydroxyisobutyrate were increased compared to exercise with high glycogen availability, suggesting increased reliance on BCAA carbon skeletons for energy production in the absence of endogenous carbohydrate stores. Data generated from this study reveal potentially increased protein requirements when endurance exercise is completed with low carbohydrate availability, taken with molecular evidence reporting increased skeletal muscle protein oxidation and breakdown (Howarth et al., 2010; Lemon & Mullin, 1980).

It is imperative to underscore that while high‐throughput metabolomics data can provide many potential insights, these findings are only as valuable as the researcher's capability to interpret them in the context of existing knowledge. A deep understanding of the literature pertinent to the research question is paramount. The researcher must possess the skill and knowledge to discern biologically relevant signals from noise. In other words, understanding the broader scientific narrative aids in identifying which metabolic changes are truly meaningful and why.

## 7 FLUXOMICS: MOVING TOWARDS A COMPLETE PICTURE OF METABOLIC REGULATION DURING EXERCISE?

The relatively few exercise metabolomics studies have deepened our understanding of exercise metabolism, uncovering pathways associated with exercise performance and training adaptation. An improved understanding of the global biomolecular response to exercise may also allow tailored exercise and nutrition recommendations based on underlying metabolic profiles on any given day. Despite the potential of metabolomics, due to the rapid, dynamic nature of metabolism, resolving the origin and fate of specific metabolites represents a considerable pitfall of current approaches. For instance, metabolomics provides only a static metabolic snapshot of tissue or biofluid, and given the balance of production and utilisation determines metabolite concentrations, understanding the rate at which metabolites move through a metabolic pathway (i.e., metabolic flux) is essential contextual information for the interpretation of metabolomics data (Winter & Kromer, 2013). The dynamics of metabolic pathways can be understood by introducing a stable isotope tracer and subsequent measurement of labelled downstream products with a higher rate of tracer labelling, indicating higher metabolic flux.

**FIGURE 5:**
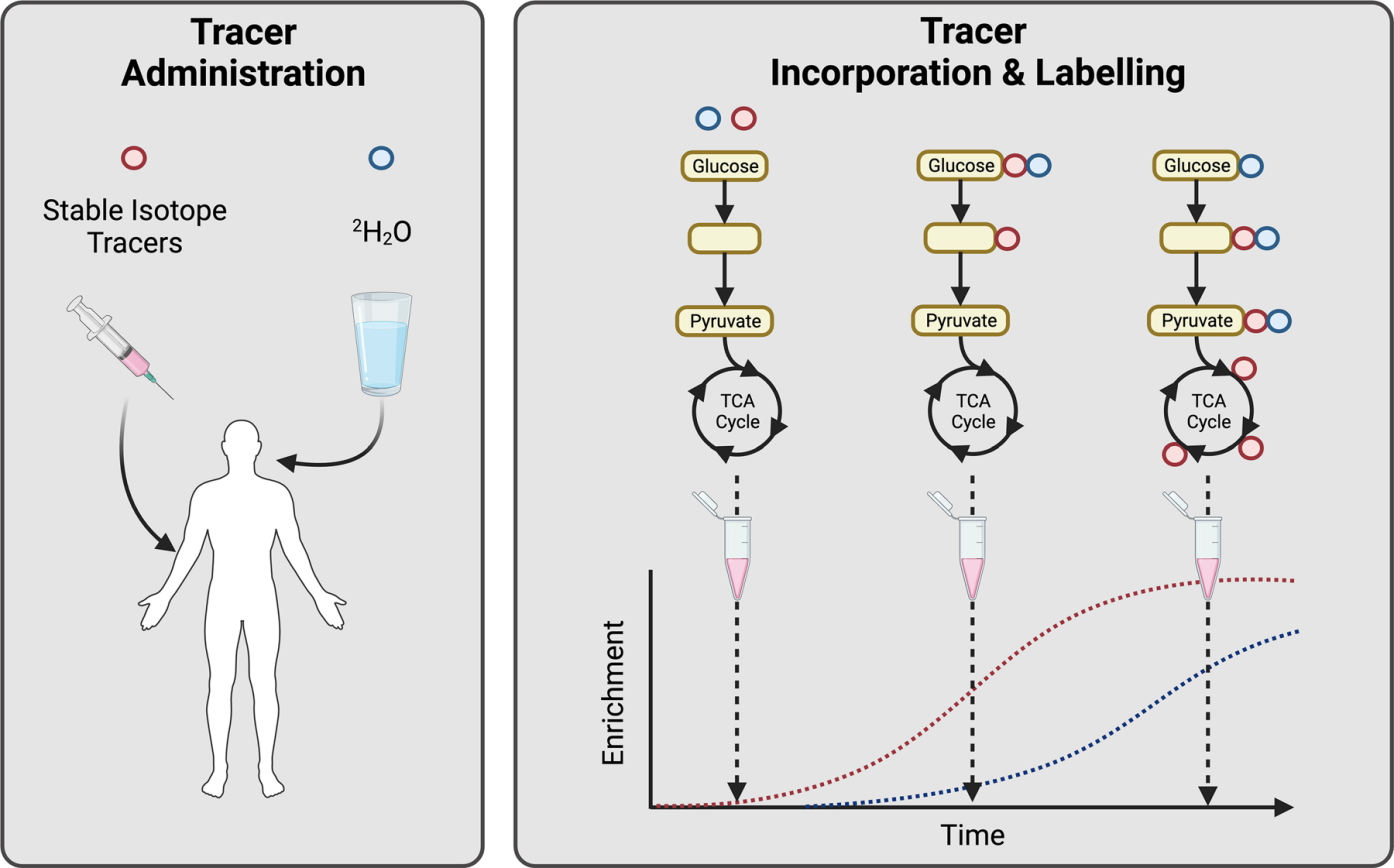
Overview of how stable isotope tracer techniques can be implemented to investigate specific pathway metabolic flux (fluxomics). Stable isotope tracers typically require intravenous administration, are highly substrate‐specific and are typically applied for short‐term metabolomics investigations. Deuterium oxide (^2^H_2_O) represents a non‐specific isotope label and is useful for longer‐duration interventions due to ease of administration (in drinking water) and the more extended labelling period required. When a stable isotope label is present, downstream metabolites are subsequently labelled with the isotopic label (enrichment), which appears in successive downstream metabolites as time series samples are collected (dependent on overall metabolic rate).

A significant progression from metabolomics, fluxomics relies on the administration of stable isotope tracers to ‘trace’ the fate of isotopically labelled downstream metabolites within specific metabolic pathways (Figure 5). Stable isotopes represent variants of an element with the same number of protons and chemical properties, making them functionally identical but with a variable number of neutrons and, therefore, mass. For example, carbon has two stable isotopes: ^12^C with a natural abundance of 98.9% and ^13^C with one additional neutron and a natural abundance of 1.1%. When introduced to a biological system via a labelled isotope tracer, the stable isotope will be incorporated into newly synthesised metabolites, which can be identified via MS. Additionally, stable isotopes are primarily substrate specific, with labelled amino acids (1,2‐^13^C_2_ leucine or ring‐^13^C_6_ phenylalanine), fatty acids (U‐^13^C palmitate) and glucose (U‐^13^C glucose), each labelling downstream metabolites in their respective metabolic pathways. While measuring specific pathway flux has been achieved in complex organisms, few studies have successfully applied multiple stable isotope tracers to measure fluxomics (Winter & Kromer, 2013). Furthermore, the application of isotopically labelled tracers in humans requires intravenous infusion, with multiple blood samples and tissue biopsies all requiring a clinical setting, restricting measures to relatively short timeframes (<24 h). Overcoming many of these limitations, non‐substrate‐specific tracers, such as deuterium dioxide (D_2_O or ^2^H_2_O), which can be administered in drinking water, permit simultaneous measurement of multiple substrates in free‐living individuals, allowing an unbiased interrogation of energy substrate flux in response to exercise.

Despite considerable advances in the application of stable isotope tracers in metabolism research, the introduction of isotope tracing techniques will greatly increase the complexity of any study from both a logistical and analytical perspective, likely requiring further technical expertise. For example, rather than measuring metabolite abundance as is the case with metabolomics, isotopic enrichment must be determined to quantify metabolic flux i.e. mass isotopomer distribution (incorporation of the isotope into a molecule) over a given time frame, by extension requiring multiple sampling time points, greater analytical resolution and mathematical modelling. For further reading on the use of isotope tracers in metabolism research, we direct readers to two reviews (Jang et al., 2018; Kim et al., 2016).

## 8 FUTURE DIRECTIONS FOR EXERCISE METABOLOMICS

Appreciating that metabolites can trigger broad downstream effects that stimulate exercise adaptation is critical. For example, the connection between muscle signalling responses and substrate utilisation during and after a single session of steady‐state cycling in well‐trained individuals who commenced exercise with either low or high muscle glycogen levels has been known for some time (Wojtaszewski et al., 2003). This study and subsequent studies on the effect of low glycogen availability, signalling and adaptive responses have primarily relied upon targeted, single‐analyte approaches. However, omics technologies can expand our understanding in such domains to capture a broader, interconnected picture of the response to nutrition and exercise metabolism. In this sense, an integrated approach that captures high throughput changes in metabolome, epigenome, transcriptome and proteome can help to connect the dots between signal and response. Indeed, the emergence of ‘multi‐omic approaches’ represents a shift in biological research towards studying biological systems holistically by integrating data from multiple omics disciplines. For example, advances have already been made in exercise immunometabolism, whereby whole‐system, metabolomics, lipidomics and proteomics have given insight into the efficacy of post‐exercise dietary interventions to support immunity (Nieman et al., 2019). Collective efforts to understand the multi‐omics response to exercise have also recently emerged. The Molecular Transducers of Physical Activity Consortium (MoTrPAC) is a large‐scale initiative funded by the National Institutes of Health (NIH) to study the molecular changes that occur during and after exercise. Several pre‐print articles have recently emerged that characterise various responses to exercise, such as the mitochondrial multi‐omic response to exercise training across tissues (Amar et al., 2022, 2023). The plasticity of the metabolome to chronic training and dietary intervention remains to be explored in detail. Long‐term training appears to affect the metabolome of athletes across different sports (Schranner et al., 2021), but the adaptability during training and detraining remains unknown. Moreover, how this regulates and is regulated by the transcriptome and proteome remains elusive.

From a technological perspective, exercise metabolomics will also benefit from improved validated metabolite databases, which increase the number of metabolites that can be confidently identified with ^1^H‐NMR and MS approaches. In the next decade, we will likely see an increase from hundreds to thousands of such validated metabolites, giving more profound insight into the metabolic pathways central to integrative exercise metabolism. Furthermore, researchers are encouraged to deposit metabolomics data (and other omics data), including metabolite structures, spectra, concentrations and raw data from metabolomics experiments, in publicly accessible repositories such as the European Bioinformatics Institute's platform Metabolights (http://www.ebi.ac.uk/metabolights) (Haug et al., 2013). The curation of metabolomics data allows researchers to more readily compare, contrast, and make inferences from their results based on the findings of others (Goodacre et al., 2004). Without considerable engagement of the metabolomics community, the advancement of the field would undoubtedly have been delayed.

## 9 CONCLUSION

Exercise metabolomics has contributed to an improved understanding of exercise metabolism and regulation of exercise adaptation, but the picture likely remains incomplete. Our current understanding of how different pathways are integrated, particularly at a multicellular level, and how they are coordinated is poorly understood (Lavin et al., 2022) and presents new opportunities for exercise physiologists to exploit approaches like metabolomics to answer these pertinent questions. It is crucial that researchers pay attention to experimental design and do not perform metabolomics as an afterthought. Similarly, planning statistical approaches during experimental design is also critical. Ultimately, exercise physiology researchers must appreciate that even a well‐performed exercise metabolomics experiment requires a deep understanding of the broader scientific narrative to discern meaningful metabolic changes.

## AUTHOR CONTRIBUTIONS

Daniel John Owens and Samuel Bennett contributed equally in the conception of this work and in the drafting of the work and revising it critically for important intellectual content. Both authors have read and approved the final version of this manuscript and agree to be accountable for all aspects of the work in ensuring that questions related to the accuracy or integrity of any part of the work are appropriately investigated and resolved. All persons designated as authors qualify for authorship, and all those who qualify for authorship are listed.

## CONFLICT OF INTEREST

The authors declare no competing interests.

## FUNDING INFORMATION

The authors received no funding to produce this review article.
